# Whole Grain Rye Intake, Reflected by a Biomarker, Is Associated with Favorable Blood Lipid Outcomes in Subjects with the Metabolic Syndrome – A Randomized Study

**DOI:** 10.1371/journal.pone.0110827

**Published:** 2014-10-23

**Authors:** Ola Kally Magnusdottir, Rikard Landberg, Ingibjorg Gunnarsdottir, Lieselotte Cloetens, Björn Åkesson, Fredrik Rosqvist, Ursula Schwab, Karl-Heinz Herzig, Janne Hukkanen, Markku J. Savolainen, Lea Brader, Kjeld Hermansen, Marjukka Kolehmainen, Kaisa Poutanen, Matti Uusitupa, Ulf Risérus, Inga Thorsdottir

**Affiliations:** 1 Unit for Nutrition Research, Landspitali -The National University Hospital of Iceland and Faculty of Food Science and Nutrition, School of Health Sciences, University of Iceland, Reykjavík, Iceland; 2 Department of Food Science, BioCenter, Swedish University of Agricultural Sciences, Uppsala, Sweden; 3 Division of Nutritional Epidemiology, Institute of Environmental Medicine, Karolinska Institutet, Stockholm, Sweden; 4 Biomedical Nutrition, Pure and Applied Biochemistry, Lund University, Lund, Sweden; 5 Department of Clinical Nutrition, Skåne University Hospital, Lund, Sweden; 6 Department of Public Health and Caring Sciences, Clinical Nutrition and Metabolism, Uppsala University, Uppsala, Sweden; 7 Institute of Public Health and Clinical Nutrition, University of Eastern Finland, Kuopio, Finland; 8 Institute of Clinical Medicine, Internal Medicine, Kuopio University Hospital, Kuopio, Finland; 9 Institute of Biomedicine, Department of Physiology and Biocenter, Oulu University, Oulu, Finland and Department of Psychiatry, Kuopio University Hospital, Kuopio, Finland; 10 Department of Internal Medicine and Biocenter, Institute of Clinical Medicine, University of Oulu, Oulu, Finland and Clinical Research Center, Oulu University Hospital, Oulu, Finland; 11 Department of Endocrinology and Internal Medicine, Aarhus University Hospital, Aarhus, Denmark; 12 VTT Technical Research Centre of Finland, Espoo and Kuopio, Finland; 13 Research Unit, Kuopio University Hospital, Kuopio, Finland; University of Sevilla, Spain

## Abstract

**Background and Aim:**

Few studies have explored the possible plasma cholesterol lowering effects of rye consumption. The aim of this secondary analysis in the SYSDIET study was to investigate the association between plasma alkylresorcinols (AR), a biomarker for whole grain wheat and rye intake, and blood lipid concentrations in a population with metabolic syndrome. Furthermore, we analyzed the associations between the AR C17∶0/C21∶0 ratio, a suggested marker of the relative intake of whole grain/bran rye, and blood lipid concentrations.

**Methods:**

Participants were 30–65 years of age, with body mass index (BMI) 27–40 kg/m^2^ and had metabolic syndrome. Individuals were recruited through six centers in the Nordic countries and randomized either to a healthy Nordic diet (ND, n = 93), rich in whole grain rye and wheat, as well as berries, fruits and vegetables, rapeseed oil, three fish meals per week and low-fat dairy products, or a control diet (n = 65) for 18/24 weeks. Associations between total plasma AR concentration and C17∶0/C21∶0 homologue ratio and blood lipids were investigated in pooled (ND + control group) regression analyses at 18/24 weeks adjusted for baseline value for the dependent variable, age, BMI and statin use.

**Results:**

When adjusted for confounders, total plasma AR at 18/24 weeks was not significantly associated with blood lipids but the AR ratio C17∶0/C21∶0 was inversely associated with LDL cholesterol concentrations (B (95% CI): −0.41 (−0.80 to −0.02)), log LDL/HDL cholesterol ratio (−0.20 (−0.37 to −0.03)), log non-HDL cholesterol (−0.20 (−0.37 to −0.03)), log apolipoprotein B (−0.12 (−0.24 to 0.00)) and log triglyceride concentrations (−0.35 (−0.59 to −0.12)).

**Discussion:**

Increased proportion of whole grain rye, reflected by a biomarker, in the diet is associated with favorable blood lipid outcomes, a relationship that should be further investigated.

**Trial Registration:**

ClinicalTrials.gov NCT00992641

## Introduction

Epidemiological studies have shown an inverse relationship between diets rich in whole grain and the risk of many diet-related diseases, including cardiovascular diseases [1], [2], metabolic syndrome [3] and type 2 diabetes [4]. A number of intervention studies have shown that intake of whole grains lowers serum total and LDL cholesterol concentrations [5]–[8], blood pressure [9], [10] and improves insulin sensitivity [11]–[13]. Animal studies have shown cholesterol lowering effects of rye consumption [14]–[16] but few intervention studies have studied the effects of rye on blood lipids in humans, despite the fact that whole grain rye products contain substantial amounts of extractable arabinoxylans and β-glucans [17] that may contribute to cholesterol reduction. One study has shown that rye bread consumption decreased total and LDL cholesterol concentrations in men with moderately elevated serum cholesterol [5] but another study showed an unfavorable shift in serum cholesterol concentrations after rye bread intake in postmenopausal women [18].

In nutritional science it is essential to have accurate dietary information to assess compliance to dietary interventions and to investigate diet-disease associations. Collecting valid data on whole grain intake can, however, be challenging due to the broad selection of foods contributing to these intakes [19]. The use of alkylresorcinols (AR) as dietary biomarkers for whole grain wheat and rye intake could improve the validity of dietary assessment of whole grain in combination with, or as an alternative for, self-reports [19]–[22]. It may also be used as a tool for secondary analysis, where non-compliant individuals can be excluded on the basis of the biomarker measurements. AR are 3,5-dihydroxy-phenolic lipids with an odd-numbered alkyl chain generally ranging from C17 to C25. Among commonly consumed foods, AR are almost exclusively present in the outer parts of wheat and rye grains, and just at trace amounts in refined white flour [23], [24]. The ratio of the AR homologues C17∶0 and C21∶0 differs between cereals but is consistent within cereal species (approximately 0.1 in wheat and 1 in rye) [24] and may be used to differentiate sources of cereals consumed via plasma measurements [25]. We have previously demonstrated that AR are useful biomarkers for whole grain wheat and rye intake as part of a healthy Nordic diet [26]. Furthermore, significant between-group changes in non-HDL cholesterol concentrations and LDL to HDL cholesterol ratio was found in the randomized dietary intervention with a healthy Nordic diet (SYSDIET) [27].

The present study is based on a secondary analysis of the SYSDIET intervention trial and the aim was to investigate the association between plasma AR, a biomarker for whole grain wheat and rye intake, and blood lipid concentrations in a population with metabolic syndrome (MetS) participating in a randomized dietary intervention. Furthermore, the associations between the AR C17∶0/C21∶0 ratio, a suggested marker of the relative whole grain/bran rye intake, and blood lipids were analyzed.

## Methods

The current study was a part of the SYSDIET study, a randomized controlled multicenter parallel group study with balanced randomization (1∶1) performed in six intervention centers (Kuopio and Oulu, Finland; Lund and Uppsala, Sweden; Aarhus, Denmark and Reykjavik, Iceland) examining the health effects of a healthy Nordic diet (ND) in a population with MetS. The protocol for this trial and supporting CONSORT checklist are available as supporting information; see Checklist S1 and Protocol S1.

All local ethical committees (Research Ethics Committee of the Hospital District of Northern Savo and Northern Ostrobothnia Hospital District, Oulu, Finland, Regional Ethical Review Board, Lund and The Regional Ethical Review Board in Uppsala, Sweden, Ethical Committee of Aarhus County, Denmark and The Icelandic Bioethics Committee, Iceland) approved the SYSDIET study protocol which followed the Helsinki declaration guidelines and informed written consent was obtained from all participants. The trial was registered at ClinicalTrials.gov (ClinicalTrials identifier: NCT00992641, http://clinicaltrials.gov/ct2/show/NCT00992641?term=NCT00992641&rank=1) in October 2009, once all local ethical committees had given their approval. The authors confirm that all ongoing and related trials for this intervention are registered.

The study design, inclusion and exclusion criteria, study diets, the main measurements and analyses performed at each time point and main results have previously been reported in detail [27]. The present study is a secondary analysis where the association between AR and blood lipids at the end of the intervention was examined.

### Study participants

Participants in the study were primarily recruited through advertisements in newspapers, but also from previous clinical or epidemiological trials. In brief, the inclusion criteria were age 30–65 years, body mass index (BMI) 27–40 kg/m^2^, and two diagnostic criteria for metabolic syndrome as outlined by the International Diabetes Federation [28], except type 2 diabetes.

### Study design

Participants were randomly assigned to either a healthy Nordic diet or an average Nordic (control) diet for 18 or 24 weeks. Due to financial and logistic reasons the study period was 18 weeks at 4 centers (Aarhus, Uppsala, Reykjavik and Oulu) and 24 weeks at 2 centers (Lund and Kuopio) where the intervention was initiated earlier [27]. The major visits to the study clinics were on week 0 (baseline), week 12 and week 18/24. In Kuopio and Lund, the intervention was carried out from October 2009 to June 2010, in Aarhus, from January 2010 to September 2010, in Oulu, from December 2009 to October 2010, in Reykjavik, from March 2010 to October 2010 and in Uppsala, from June 2010 to November 2010.

The randomization was done by staff in each study center as described elsewhere [26]. The staff in all study centers was trained to perform measurements in a similar way and the quality management protocol was available for all staff members. The allowed weight change during the study was aimed to be less than ±4 kg due to the isocaloric design applied.

### Study diets

The study diets were planned to be isocaloric. Nordic nutrition recommendations formed the basis of the diet in the ND group [29] and the mean nutrient intake in the Nordic countries formed the basis for the control diet. Participants in the ND group were advised to consume ≥25% of total energy as whole grain, of which ≥50% was comprised of rye, barley and oat which included ≥6 slices/day of bread with ≥6 g fiber/100 g (one slice equals 30–40 g of bread). Participants in the control group were asked to consume ≥25% of energy as refined grain, of which ≥90% made from wheat. Furthermore, the participants in the ND group were encouraged to consume ≥500 g of fruits, vegetables and berries daily, use rapeseed and/or sunflower and/or soybean oil based margarines, choose low fat milk and dairy products, consume three fish meals a week, choose low fat meat, and to avoid sugar-sweetened beverages while the control group was advised to keep their habitual diet. Key food products were provided to the study participants in both groups. The individuals in the ND group received whole grain products such as oatmeal and barley, crisp breads, whole grain wheat and rye breads. The individuals in the control group received low fiber cereal products, e.g. breads with fiber content less than 6 g/100 g and ordinary pasta. The study diets have previously been described in detail [27].

The participants were instructed by a clinical nutritionist or a dietitian at all study visits. The study participants kept dietary records regarding the intake of cereal products. These records were checked at each visit to the study center, including the visits when the groceries were delivered to the participants, i.e. at 1–2 week intervals and the subjects also returned 4-day weighed food records at baseline, week 2, 12 and 18/24 [27].

### Biochemical measurements

Blood samples were collected at week 0, 12 and 18/24. Automated clinical chemistry analyzers and routine clinical chemistry methods were used to measure concentrations of total and HDL cholesterol, triglycerides and apoplipoproteins A1 and B. The equipment and system reagents and CV%’s have been described previously [27]. LDL cholesterol concentrations were calculated using Friedewald's formula.

Determination of AR in plasma samples were made in collaboration with the HELGA-Centre at Department of Food Science, BioCenter at Swedish University of Agricultural Sciences. A gas chromatography mass spectrometry (GC-MS) method was used to quantify AR homologues C17∶0−C25∶0 in 0.2 ml plasma samples as described previously [30]. Quality control samples (n = 4) was included in each batch to ensure appropriate within- and between batch precision (CV <15%). Samples from each subject were analyzed within the same batch and samples from different centers were allocated randomly between batches.

### Data pooling and statistical analysis

All intervention centers entered their data into Microsoft Office Excel 2007 (Windows) data form prior to pooling the data in a joint database maintained at VTT Technical Research Centre of Finland. Data was exported from the database and imported to SPSS (Statistical Package for the Social Sciences) for Windows, version 20.0 (SPSS Inc., Chicago, Illinois, USA) for statistical analysis. Level of significance in all analyses was set at P<0.05.

Normality of variables was checked by inspection and by using the Kolmogorov–Smirnov test. Values are described as means and standard deviations (SD) if normally distributed or as medians and interquartile range (IQR) if not normally distributed.

The analyses were performed with data from the ND and control groups combined and associations between total AR and the AR C17∶0/C21∶0 ratio at 18/24 weeks and various blood lipid outcomes were analyzed. Where distributions were skewed (every variable except total and LDL cholesterol concentrations), log-transformation was performed on dependent variables for linear regression to better approximate normal distribution. The distribution of AR C17∶0/C21∶0 ratio across total plasma AR was checked by inspection.

Linear regressions for fasting concentrations of total, LDL, HDL, non-HDL cholesterol, apolipoproteins A1 and B, triglycerides and LDL/HDL cholesterol ratio with total plasma AR or AR C17∶0/C21∶0 ratio (model 1), baseline value for the dependent variable, age, BMI and statin use, as factors were performed. As rye often contains more AR than wheat, total plasma AR was added as a variable, in addition to the variables in model 1 when the associations between AR C17∶0/C21∶0 ratio was examined, to prevent confounding of high AR content (model 2). To account for multiple testing, we used the Bonferroni correction approach, using a significance level of alpha divided by the number of independent outcomes, that is 0.05/8 = 0.006. Furthermore, we individually adjusted for saturated fat intake calculated from food records and serum phospholipid fatty acids myristic acid, palmitic acid, omega-3 fatty acids and C15∶0, to prevent confounding of dietary fat intake.

## Results

**Figure 1:**
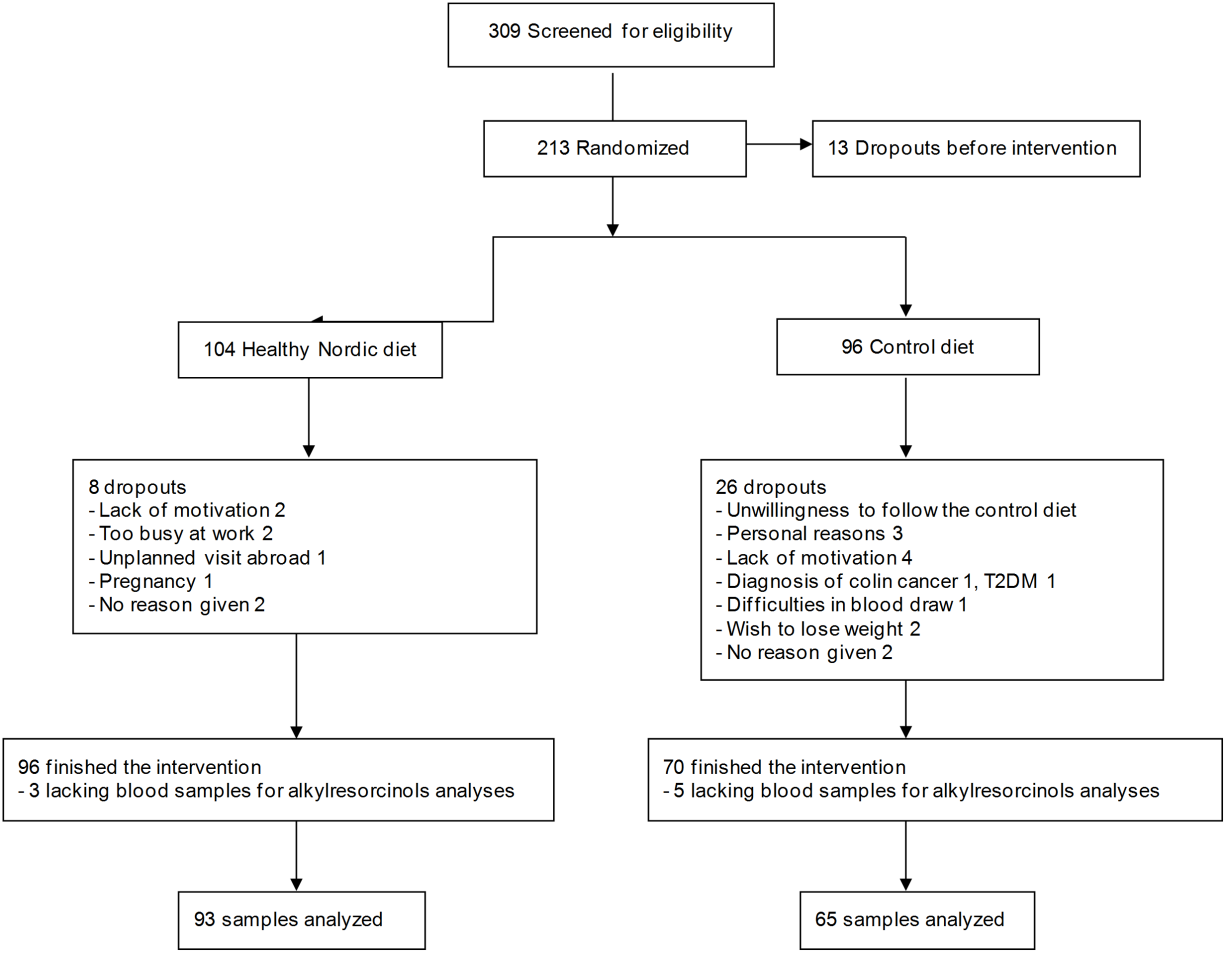
Flow chart of the study.

Altogether 166 participants finished the study and of those 158 subjects had blood samples for AR and blood lipid analyses from all time points and that group was used for analyses in the present study (Figure 1). There was no difference between dropouts and those who completed the study in baseline BMI (P = 0.797), waist circumference (P = 0.228), age (P = 0.959) or triglyceride (P = 0.055), total cholesterol (P = 0.743), HDL cholesterol (P = 0.216) or LDL cholesterol (P  = 0.686) concentrations.

There was no change in total energy intake during the study and energy intake was not different between the groups, hence, body weight remained constant during the intervention in both groups. The mean age of the participants was 54.5 (8.2) years, body weight 91.2 (13.5) kg, BMI 31.7 (3.2) kg/m^2^ and waist circumference 104.3 (9.7) cm as described previously [31]. Baseline lipid concentrations and the number of statin users in by intervention group, are shown in Table 1. The prevalence of MetS was 90%. No differences were observed between the groups at baseline in clinical characteristics.

**Table 1:** Baseline blood lipid concentrations and statin use of the participants.

	ND group	Control group
	(n = 93)	(n = 65)
Total cholesterol, *mmol/L*	5.3 (1.2)	5.1 (1.5)
HDL cholesterol, *mmol/L*	1.4 (0.5)	1.3 (0.6)
LDL cholesterol, *mmol/L*	3.2 (1.1)	3.1 (1.5)
Non-HDL cholesterol, *mmol/L*	3.9 (1.3)	3.7 (1.6)
LDL-C/HDL-C	2.5 (1.4)	2.4 (1.8)
Triglycerides 0 min, *mmol/L*	1.3 (0.8)	1.4 (0.8)
Apolipoprotein A1, *g/L*	1.4 (0.3)	1.4 (0.3)
Apolipoprotein B, *g/L*	1.0 (0.4)	1.0 (0.4)
Statin use, *n*	19	18

Values are presented as medians (IQR).

Abbreviations: HDL, high-density lipoprotein; LDL, low-density lipoprotein.

There were small, but significant, decrease in concentrations of total cholesterol (5.1 (1.2) mmol/L at 18/24 weeks, P = 0.018), LDL (3.2 (1.1) mmol/L at 18/24 weeks, P = 0.006) and non-HDL cholesterol (3.8 (1.3) mmol/L at 18/24 weeks, P = 0.006) and LDL/HDL cholesterol ratio (2.3 (1.3) at 18/24 weeks, P = 0.003) within the ND group from week 0 to 18/24 but not within the control group. Results on the effects of the SYSDIET intervention, with ND, on blood lipid concentrations and other health outcomes have been reported previously [27]. Both total plasma AR concentration and the ratio of C17∶0/C21∶0 were significantly higher in the ND group than in the control group at 18/24 week (Table 2) and the range of total plasma AR was 14 to 499 nmol/L. There was no skew towards higher AR C17∶0/C21∶0 ratios for higher total plasma AR concentrations.

**Table 2:** Dietary fiber intake, total plasma alkylresorcinols (AR) concentration and AR C17∶0/C21∶0 ratio for the healthy Nordic diet group (ND) and control group at baseline and after the 18/24 week intervention.

	ND group (n = 93)		Control group (n = 65)	
	Week 0	Week 18/24	Week 0	Week 18/24
Dietary fiber intake^1^^,^ 2, *g/day*	21.1 (8.8)	36.0 (15.1)#	20.0 (7.1)	16.0 (6.2)* #
Total plasma AR^1^, *nmol/L*	73 (88)	106 (108)#	71 (70)	61 (40)* #
C17∶0/C21∶0 ratio^1^	0.3±0.2	0.3±0.2	0.3±0.2	0.2±0.2* #

Values are medians (IQR).

*significantly different from ND group at that time point.

# significantly different from baseline.

1 Data published previously [31].

2 n = 140, ND group n = 80 and control group n = 40.

Abbreviations: ND, healthy Nordic diet; AR, alkylresorcinols.

All participants (both ND and control groups) were combined for secondary analyses on the associations between AR and blood lipids concentrations at 18/24 weeks. Total plasma AR concentration at 18/24 weeks was not significantly correlated with concentrations of fasting serum total cholesterol (P = 0.95), LDL cholesterol (P = 0.23), HDL cholesterol (P = 0.25), LDL-C/HDL-C ratio (P = 0.13), triglycerides (P = 0.14), apolipoprotein A1 (P = 0.09) or apolipoprotein B (P = 0.86) after adjusting for confounders. However, the AR C17∶0/C21∶0 ratio, which reflects the proportion of whole grain rye of whole grain rye and whole grain wheat combined, was inversely associated with LDL cholesterol concentration when adjusted for baseline LDL cholesterol concentration, BMI, age and statin use (model 1, P = 0.040) and also when total plasma AR was added to the regression model (model 2, P = 0.046) (Table 3).

**Table 3:** Regression models for concentrations of serum total, HDL, LDL, non HDL cholesterol and triglycerides and the cholesterol LDL-C/HDL-C ratio.

Dependent variable at 18/24 weeks	Independent variable at 18/24 weeks	B^1^ (95% CI)
Total cholesterol		
Model 1	AR C17∶0/C21∶0	−0.33 (−0.78 to 0.12)
Model 2	AR C17∶0/C21∶0	−0.33 (−0.78 to 0.12)
LDL cholesterol		
Model 1	AR C17∶0/C21∶0	−0.41 (−0.80 to −0.02)
Model 2	AR C17∶0/C21∶0	−0.40 (−0.79 to −0.01)
Log HDL cholesterol		
Model 1	AR C17∶0/C21∶0	0.11 (0.00 to 0.21)
Model 2	AR C17∶0/C21∶0	0.11 (0.00 to 0.21)
Log LDL-C/HDL-C cholesterol		
Model 1	AR C17∶0/C21∶0	−0.20 (−0.37 to −0.03)
Model 2	AR C17∶0/C21∶0	−0.20 (−0.37 to −0.03)
Log non HDL cholesterol		
Model 1	AR C17∶0/C21∶0	−0.84 (−1.22 to −0.45)
Model 2	AR C17∶0/C21∶0	−0.81 (−1.19 to −0.44)
Log triglycerides		
Model 1	AR C17∶0/C21∶0	−0.35 (−0.59 to −0.12)
Model 2	AR C17∶0/C21∶0	−0.37 (−0.60 to −0.13)
Log apolipoprotein A1		
Model 1	AR C17∶0/C21∶0	0.05 (−0.03 to 0.13)
Model 2	AR C17∶0/C21∶0	0.05 (−0.03 to 0.13)
Log apoliprotein B		
Model 1	AR C17∶0/C21∶0	−0.12 (−0.24 to 0.00)
Model 2	AR C17∶0/C21∶0	−0.12 (−0.24 to 0.00)

1 B reflects the change in the log-transformed outcome variable for each 1-unit changes in the independent variable.

Model 1: adjusted for baseline value of the dependent variable, BMI, age and statin use.

Model 2: adjusted for baseline value of the dependent variable, total AR, BMI, age and statin use.

Abbreviations: ND, healthy Nordic diet; AR, alkylresorcinols; HDL, high-density lipoprotein; LDL, low-density lipoprotein; BMI, body mass index.

Similarly, AR C17∶0/C21∶0 ratio was inversely associated with LDL/HDL cholesterol ratio (P = 0.020 and P = 0.024 for model 1 and model 2, respectively), non-HDL cholesterol (P<0.001 for both model 1 and model 2), triglyceride (P = 0.004 and P = 0.002 for model 1 and model 2, respectively) and apolipoprotein B concentrations (P = 0.047 for both model 1 and model 2) and positively associated with HDL cholesterol concentration (P = 0.044 and P = 0.049 for model 1 and model 2, respectively). Including saturated fatty acids calculated from food records to the models did not change the results nor did including the serum phospholipid fatty acids myristic acid, palmitic acid, omega-3 fatty acids and C15∶0, except for HDL cholesterol in which the association with C17∶0/C21∶0 became borderline significant (P = 0.067). However, using the Bonferroni correction approach to account for multiple testing the associations for LDL cholesterol, HDL cholesterol, LDL/HDL cholesterol ratio and apolipoprotein B concentrations would not be considered significant while associations for non-HDL cholesterol and triglyceride concentration are robust for this correction (P<0.006).

## Discussion

Favorable changes in serum LDL cholesterol concentration and LDL to HDL cholesterol ratio were observed in participants consuming a healthy Nordic diet, established with a healthy dietary pattern approach, and thus also rich in whole grain cereals, for either 18 or 24 weeks [27]. In the intervention the participants in the ND group were advised to consume ≥25% of total energy as whole grain, of which ≥50% was comprised of rye, barley and oat, and to make favorable changes in dietary fat intake, and therefore a change in serum cholesterol concentration in the ND group was expected. Some studies have found that whole grain diets lower serum total and LDL cholesterol concentrations [5]–[8]. The cholesterol lowering effects of β-glucans in oats and barley have been extensively studied [32], [33] and the European Food Safety Authority (EFSA) has approved two health claims on the subject: β-glucans contribute to the maintenance of normal blood cholesterol levels [34] and oat β-glucan has been shown to lower/reduce blood cholesterol. Blood cholesterol lowering by increased oats intake may reduce the risk of (coronary) heart disease [35].

In the present study, both ND and control groups were pooled for secondary analysis on the association between plasma AR and blood lipids at 18/24 weeks. Plasma AR has been established as a biomarker for whole grain wheat and rye intake [19], [21], [26] and has previously been studied regarding associations with BMI and metabolic risk factors [36], [37]. We observed no association between total plasma AR concentration and serum lipid concentrations, possibly due to a considerable amount of AR intake originating from whole grain wheat. Whole grain wheat intake does typically not affect blood cholesterol and is thus often used as a control in experimental studies [38]. Interestingly, the AR C17∶0/C21∶0 ratio, an indicator of relative rye intake, was associated with favorable changes in concentrations of LDL-, HDL-, and non HDL cholesterol, LDL/HDL cholesterol ratio and triglycerides, even after adjustment for fatty acids composition of serum phospholipids, with the exception of HDL cholesterol where the association became borderline significant. The ratio of the AR homologues C17∶0 and C21∶0 differs between cereal species and can be used to differentiate between the whole grain sources in food products [24]. The ratio of AR C17∶0/C21∶0 is approximately 0.1 in wheat and 1 in rye and the ratio is to some extent reflected in fasting plasma samples, although it is generally lower, typically around 0.6–0.8 in fasting samples after a whole grain rye intervention for 1–8 weeks, and can be used to differentiate which AR containing cereals have been consumed [25], [39], [40].

Health effects of rye are far less studied than oats and barley and studies on the effects of rye consumption on cholesterol are scarce. The mechanisms that have been proposed to cause the reduction in plasma cholesterol levels focus on the fiber and phytochemical content of whole grains, particularly water-soluble viscose fiber [41], [42], and it has been established that the cholesterol lowering effect of β-glucans is dependent on the molecular weight and subsequent viscosity [43]. Rye is particularly high in dietary fiber, ranging between 18–22%, and it contains substantial amounts of extractable β-glucans and arabinoxylans, which could contribute to cholesterol reduction through a similar mechanism as β-glucans from oats and barley [17], [44]. Animal studies have shown that rye could affect blood lipid concentrations by increasing the total concentration of bile acids in the bile [14]–[15].

Previous studies have shown that rye bread consumption decreases total and LDL cholesterol concentrations in men with moderately elevated serum cholesterol [5], and lower concentrations of plasma free-cholesterol, total cholesterol, triglycerides and phospholipids have been demonstrated after a high fiber rye diet than a low fiber wheat diet [6]. Additionally, animal studies have shown cholesterol lowering effects of rye consumption [14]–[16]. However, Moazzami et al showed an unfavorable shift in serum cholesterol levels after rye bread intake in postmenopausal women [18]. Processing of rye and other whole grains, such as fermentation, have been reported to influence the molecular weight of cereal dietary fiber, hence it is possible that lower viscosity of the fibers could explain the lack of beneficial effects reported in some studies [17]. Nevertheless, the results of the present study suggest that whole grain rye consumption is associated with favorable outcomes in blood lipids and this association should be further studied.

One of the strengths of the present study is that we used AR as a dietary biomarker for whole grain rye and wheat intake instead of self-reported estimates. As collecting valid data on whole grain intake can be challenging, the use of biomarkers to assess intake and/or compliance to dietary interventions is a way to avoid some of the obstacles associated with self-reporting [19]. Previously, we have demonstrated that AR is a good biomarker for the ND under intervention conditions with moderate to good reliability [26] and our data supports the use of the AR C17∶0/C21∶0 ratio as an indicator for whole grain rye intake in intervention studies where a diet rich in whole grain rye is being investigated [31]. Although the AR C17∶0/C21∶0 ratio reflects only the relative whole grain/bran rye consumption of total whole grain wheat and rye intake, and not the total rye intake, the advised whole grain rye intake in the present study was high in relation to other sources of AR and therefore the ratio should reflect the total rye intake reasonably well. However, the present study does not offer any mechanistic explanations behind the possible cholesterol-lowering by whole grain rye, and more needs to be done to explain the association. Nevertheless, the results do motivate further research on this topic. Some confounding effects from oats intake are possible, as participants consuming more rye might also consume more oats, and confounding effects from other food sources cannot be excluded. However, this is probably not explaining the main results. Previous studies suggest that plasma AR may be transported in VLDL and HDL [45], which could affect the results of the present study, as well as numerous dietary and non-dietary factors potentially influencing biomarker concentrations [19]. Nevertheless, since the AR C17∶0/C21∶0 ratio, but not total plasma AR, was associated with cholesterol concentrations, and the associations did not change when adjusted for total plasma AR, we believe our findings indicate that the associations we found are related to whole grain rye intake and not by the fact that AR may be transported in certain lipoproteins.

## Conclusion

Our findings suggest that a greater proportion of whole grain rye in a Nordic diet is associated with favorable outcomes in blood lipid concentrations, an association that warrants further investigation.

## Supporting Information

Checklist S1 **Consort 2010 checklist of information to include when reporting a randomized trial.**
(DOC)

Protocol S1 **SYSDIET study plan.**
(DOC)
