# Supplementary material for: Whole Grain Rye Intake, Reflected by a Biomarker, Is Associated with Favorable Blood Lipid Outcomes in Subjects with the Metabolic Syndrome – A Randomized Study
Source: PLoS One. 2014 Oct 23;9(10):e110827. doi: 10.1371/journal.pone.0110827 (PMC4207773; doi:10.1371/journal.pone.0110827)
Supplement: Checklist S1 — Consort 2010 checklist of information to include when reporting a randomized trial. (DOC) [file pone.0110827.s001.doc]

Checklist S1. Consort 2010 checklist of information to include when reporting a randomized trial

| Section/Topic | Item No | Checklist item | Reported in section |
| --- | --- | --- | --- |
| Title and abstract | | | |
|  | 1a | Identification as a randomised trial in the title | Title |
|  | 1b | Structured summary of trial design, methods, results, and conclusions (for specific guidance see CONSORT for abstracts) | Abstract |
| Introduction | | | |
| Background and objectives | 2a | Scientific background and explanation of rationale | Introduction |
|  | 2b | Specific objectives or hypotheses | Introduction |
| Methods | | | |
| Trial design | 3a | Description of trial design (such as parallel, factorial) including allocation ratio | Methods |
|  | 3b | Important changes to methods after trial commencement (such as eligibility criteria), with reasons | Methods – Study design and Uusitupa et al, 2013, pages 53-54 |
| Participants | 4a | Eligibility criteria for participants | Methods – Study participants and Uusitupa et al, 2013, pages 54-55 |
|  | 4b | Settings and locations where the data were collected | Methods – Study design |
| Interventions | 5 | The interventions for each group with sufficient details to allow replication, including how and when they were actually administered | Methods – Study design and Methods – Study diets and Uusitupa et al, 2013, pages 53-57 |
| Outcomes | 6a | Completely defined pre-specified primary and secondary outcome measures, including how and when they were assessed | Methods – Biochemical measurements |
|  | 6b | Any changes to trial outcomes after the trial commenced, with reasons | N/A |
| Sample size | 7a | How sample size was determined | Uusitupa et al, 2013, page 57 |
|  | 7b | When applicable, explanation of any interim analyses and stopping guidelines | N/A |
| Randomisation: |  |  |  |
| Sequence generation | 8a | Method used to generate the random allocation sequence | Uusitupa et al, 2013, page 53 |
|  | 8b | Type of randomisation; details of any restriction (such as blocking and block size) | Uusitupa et al, 2013, page 53 |
| Allocation concealment mechanism | 9 | Mechanism used to implement the random allocation sequence (such as sequentially numbered containers), describing any steps taken to conceal the sequence until interventions were assigned | Uusitupa et al, 2013, page 53 |
| Implementation | 10 | Who generated the random allocation sequence, who enrolled participants, and who assigned participants to interventions | Methods – Study design and Uusitupa et al, 2013, page 53 |
| Blinding | 11a | If done, who was blinded after assignment to interventions (for example, participants, care providers, those assessing outcomes) and how | N/A |
|  | 11b | If relevant, description of the similarity of interventions | N/A |
| Statistical methods | 12a | Statistical methods used to compare groups for primary and secondary outcomes | Methods - Data pooling and statistical analysis |
|  | 12b | Methods for additional analyses, such as subgroup analyses and adjusted analyses | Methods - Data pooling and statistical analysis |
| Results | | | |
| Participant flow (a diagram is strongly recommended) | 13a | For each group, the numbers of participants who were randomly assigned, received intended treatment, and were analysed for the primary outcome | Results |
|  | 13b | For each group, losses and exclusions after randomisation, together with reasons | Results, Figure 1 |
| Recruitment | 14a | Dates defining the periods of recruitment and follow-up | Uusitupa et al, 2013, page 54 |
|  | 14b | Why the trial ended or was stopped | N/A |
| Baseline data | 15 | A table showing baseline demographic and clinical characteristics for each group | Uusitupa et al, 2013, page 58 |
| Numbers analysed | 16 | For each group, number of participants (denominator) included in each analysis and whether the analysis was by original assigned groups | Results, Tables 1-3 |
| Outcomes and estimation | 17a | For each primary and secondary outcome, results for each group, and the estimated effect size and its precision (such as 95% confidence interval) | Results, Tables 1-3 |
|  | 17b | For binary outcomes, presentation of both absolute and relative effect sizes is recommended | N/A |
| Ancillary analyses | 18 | Results of any other analyses performed, including subgroup analyses and adjusted analyses, distinguishing pre-specified from exploratory | Results, Tables 1-3 |
| Harms | 19 | All important harms or unintended effects in each group (for specific guidance see CONSORT for harms) | N/A |
| Discussion | | | |
| Limitations | 20 | Trial limitations, addressing sources of potential bias, imprecision, and, if relevant, multiplicity of analyses | Discussion |
| Generalisability | 21 | Generalisability (external validity, applicability) of the trial findings | Discussion |
| Interpretation | 22 | Interpretation consistent with results, balancing benefits and harms, and considering other relevant evidence | Discussion |
| Other information | | |  |
| Registration | 23 | Registration number and name of trial registry | Abstract and Methods |
| Protocol | 24 | Where the full trial protocol can be accessed, if available | Uusitupa et al, 2013, ClinicalTrials.gov identifier: NCT009926 |
| Funding | 25 | Sources of funding and other support (such as supply of drugs), role of funders | Funding |

**References**

Uusitupa M, Hermansen K, Savolainen MJ, Schwab U, Kolehmainen M, Brader L, et al. Effects of an isocaloric healthy Nordic diet on insulin sensitivity, lipid profile and inflammation markers in metabolic syndrome -- a randomized study (SYSDIET). Journal of internal medicine. 2013;274(1):52-66.
