# Supplementary material for: Whole Grain Rye Intake, Reflected by a Biomarker, Is Associated with Favorable Blood Lipid Outcomes in Subjects with the Metabolic Syndrome – A Randomized Study
Source: PLoS One. 2014 Oct 23;9(10):e110827. doi: 10.1371/journal.pone.0110827 (PMC4207773; doi:10.1371/journal.pone.0110827)
Supplement: Protocol S1 — SYSDIET study plan. (DOC) [file pone.0110827.s002.doc]

**Protocol S1.** SYSDIET study plan

**Healthy Nordic Diet in the Prevention of Metabolic Syndrome**

**A multi-centre study of the effects of healthy Nordic food in persons with features of metabolic syndrome**

**ClinicalTrials.gov Identifier: NCT00992641**

Matti Uusitupa et al. for the Nordic Centre of Excellence SYSDIET consortium

*** Amendments to the original plan have been marked with red writing.**

1. **Background**

SYSDIET (Systems biology in controlled dietary interventions and cohort studies) is one of the three centres in the NCoE Food, Nutrition and Health, 2007-2011. It consists of 12 partners from five Nordic countries working on multidisciplinary fields of science related to nutritional biology. The main objective of SYSDIET is to reveal mechanisms by which Nordic foods and diets could be modified to promote health and prevent insulin resistance, type 2 diabetes and cardiovascular diseases, all of which being connected to metabolic syndrome. Furthermore, the aim is to build up a Nordic platform for cohort studies and carefully conducted multi-centre dietary intervention studies, where novel nutritional systems biology tools can be applied besides human studies also in animal and cell culture studies.

In order to achieve the main objective a Nordic multi-centre randomized controlled human intervention study is being conducted in 2009-2010 in 6-8 centres of SYSDIET consortium. In the following, a condensed study plan is presented for this dietary intervention study aiming primarily at finding out the effects of healthy Nordic food in persons with features of metabolic syndrome. This study plan is based on the discussion of the SYSDIET consortium in Oslo, April, 10-11, 2008.

Health of the Nordic populations has substantially improved during the last 30 years. This is due e.g. to marked decline in cardiovascular morbidity and mortality. However, during the last 10-20 years increasing obesity and sedentary lifestyle have resulted in an increase of metabolic syndrome and type 2 diabetes. This concerns not only middle-aged people but also children and adolescents. The prevalence of metabolic syndrome is 15 to 36 % in different study populations among middle-aged and elderly people, but figures may vary among different nations. It has been suggested that the epidemic of obesity/metabolic syndrome/type 2 diabetes may arrest the beneficial development of a nation’s health achieved within the last decades in Nordic countries. Therefore, we decided in the SYSDIET consortium to focus our research interest on metabolic syndrome and its consequences and especially on the prevention and treatment of metabolic syndrome by changes diet according to Nordic recommendations from 2004. Furthermore, in this consortium we aim to find out early biomarkers, which are related to the development of metabolic syndrome. These biomarkers could be originated from our diet or they may be “new” metabolites linked to the risk of metabolic syndrome. Along with this line, systems biology approaches are being applied in our studies on diet and metabolic syndrome.

Metabolic syndrome can be described as clustering of risk factors, which links it to an increased risk of type 2 diabetes and atherosclerotic vascular diseases. Several criteria have been created as to definition of metabolic syndrome. Recently central obesity has been considered as a new key feature, due to its simplicity to measure. The International Diabetes federation criteria for metabolic syndrome have been listed in Table 1.

The main risk factors of metabolic syndrome and type 2 diabetes are obesity, in particular central obesity, and sedentary lifestyle. In addition, overall quality of diet, diet rich in fat and saturated fatty acids, high glycaemic index carbohydrate diet as well as diet low in dietary fibre have been shown to associate with the risk of these conditions. In some epidemiological studies fish intake has been shown to be protective in terms of the risk of diabetes, but short-term human intervention trials have not confirmed the relation between glucose and insulin metabolism and fish or omega-3-fatty acid intake. Omega-3-fatty acids have many other beneficial effects, which could be of interest regarding clinical features of metabolic syndrome and the risk of atherosclerotic vascular diseases. Interestingly, fish protein is recently suggested to have lowering effects on blood pressure, but this finding should be confirmed by further studies.

**Table 1.** Criteria of the metabolic syndrome by International Diabetes Federation.

| **Primary Criterion:** |  |
| --- | --- |
| Central Obesity | Waist circumference - ethnicity specific: men >94 cm, women: >80 cm |
|  |  |
| **Plus any two:** |  |
| Raised triglycerides | >150 mg/dL (1.7 mmol/L) |
|  | Specific treatment for this lipid abnormality |
|  |  |
| Reduced HDL-cholesterol | <40 mg/dL (1.03 mmol/L) in men  <50 mg/dL (1.29 mmol/L) in women |
|  | Specific treatment for this lipid abnormality |
|  |  |
| Raised blood pressure | Systolic 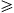130 mm Hg  Diastolic 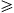85 mm Hg |
|  | Treatment of previously diagnosed hypertension |
|  |  |
| Raised fasting plasma glucose | Fasting plasma glucose 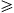100 mg/dL (5.6 mmol/L)  Previously diagnosed type 2 diabetes |

(Alberti KGMM et al. The metabolic syndrome - a new worldwide definition, Lancet 2005)

Whole grain cereal products, fruit, berries and vegetables have been shown to be protective against many chronic diseases. The consumption of whole grain cereal products, fruit, berries and vegetables has been promoted by various recommendations including Nordic recommendations from 2004 (Nordic Nutrition Recommendations 2004, 4^th^ edition, NORD 2004:13). Mediterranean diet has gained a lot of interest due to its health benefits. The key dietary items in this health promoting diet are fruit, vegetables, fish and olive oil. Both epidemiological and some dietary intervention studies have shown that Mediterranean diet may protect against cardiovascular diseases. More specifically, olive oil, which is rich in monounsaturated fatty acids, is beneficial also in terms of lipid and glucose metabolism.

The aim of the Nordic Dietary recommendations is to promote public health integrating nutrition and physical activity. These recommendations are based on solid scientific evidence with regard to prevent chronic diseases, e.g. atherosclerotic vascular diseases, obesity, metabolic syndrome and type 2 diabetes. In principle, the composition of Nordic healthy food is in line with other dietary recommendations. They emphasize energy balance, restriction of the intake of saturated fats, moderate intake of unsaturated fats, increased use of dietary fibre, fruit, berries, vegetables and whole grain cereal products, and use of low fat dairy products. Furthermore, salt intake should be reduced in long-term. As for fish intake, regular consumption of fish, both fatty and lean varieties, is recommended. This means two fish meals per week.

Based on recent findings e.g. from Finland, persons with metabolic syndrome have poorer quality of diet as compared to people without this condition. Thus, dietary patterns are differentiating people in relation to their health status. Several long-term intervention trials, e.g. the Finnish Diabetes Prevention Study, show that weight reduction, moderate increase in physical activity and beneficial dietary changes according to current recommendations result in the decrease in the risk of type 2 diabetes and metabolic syndrome. However, it has remained an open question as for what is the role of changes in the quality of diet without simultaneous weight loss and increased physical activity. More importantly, there are no studies available testing whether a healthy Nordic diet could reduce the main biochemical and physiologic abnormalities of metabolic syndrome. Furthermore, Nordic food includes many other ingredients which may be beneficial in the prevention of chronic diseases. In particular phytochemicals are gaining increasing interest due to their potential health effects. Therefore, more information is urgently needed in order to explore the health potential of healthy Nordic foods in promotion of health and prevention and treatment of chronic diseases, especially metabolic syndrome.

1. **Aims of the study**

Having this background, the aim of the SYSDIET consortium is to carry out a controlled, randomized dietary intervention study in persons with features of metabolic syndrome to find out the effects of a healthy Nordic food on major abnormalities in metabolic syndrome.

**Specific objectives:**

1. To find out to what extent a Nordic healthy food could improve insulin sensitivity and other metabolic/physiological abnormalities in persons with features of metabolic syndrome
2. To identify new early markers for insulin resistance/metabolic syndrome in serum and urine
3. To study the effects of the healthy Nordic food on gene expression in adipose (AT) tissue and peripheral blood mononuclear cells (PBMCs)
4. To study the effects of the healthy Nordic food on lipid and metabolite profiles (metabolomics)
5. To examine how healthy Nordic food may effect on gut flora
6. To build up Nordic consortium (platform) to carry out controlled dietary intervention studies to make Nordic countries more competitive in food science and human nutrition studies

When it comes to metabolic syndrome several outcome measures are to be considered. Nevertheless, insulin resistance is a characteristic feature in this condition. Therefore, the main outcome measures will be insulin resistance index and insulin secretion index both of which are based on oral glucose tolerance test (OGTT) with 0, 30 min, and 120 min glucose, insulin and triglyceride values.

1. **Study design**

The study design is described in the Figure 1. Recruited persons will start the study by following their conventional diet for one month as a run-in period. After that subjects will be randomly assigned into Experimental- (Exp) or Control- diet-group for 6 months. They will visit the study clinic at 2, 4, 8, 12, 16, 20 and 24 weeks. At each visit body weight and BP will be measured. The major visits are in the beginning (0 week) and at 12 and 24 weeks (end of the study), and will be done after 12 hour fasting. Subjects should drink 2.5 dl water both in the evening and in the morning during the fasting period before the study visits. Staff in all study centers will be trained to perform the measurements and quality management protocol. Standard operational procedures will be followed in all measurements.

**The original protocol was changed after the discussion with the Food, Nutrition and Health –program Scientific Advisory Board and Steering Group members due the following reasons:**

**1. A shorter period of intervention will give the same information as that obtained from 24 weeks trial.**

**2. Total costs will be lower and recruitment of subjects easier to carry out.**

**3. Analyses will be started earlier in September 2010.**

**Therefore, it was decided to shorten the intervention to 18 weeks in Århus, Uppsala, Reykjavik and Oulu. This means that the last visit will happen at week 18, but otherwise the visits to the study centres will happen according to original study plan. In the analyses of the major outcome measures no changes are allowed (see below).**

Because physical activity, alcohol intake, smoking, body weight and drugs are known to affect the characteristics of metabolic syndrome, the study persons are advised to keep physical activity constant, and not to change their smoking and drinking habits or drug treatment during the study.

Planning phase of the study was started in 2008 and the trial will be carried out in 2009-2010.

- 1. **Screening**

At the first visit 1 month before the study a screening examination will be carried out. The visit includes medical history, clinical examination, body weight, height, waist circumference, blood pressure, blood count, fasting plasma glucose and fasting serum creatinine, tyreotropin stimulating hormone (TSH), gamma-GT, AFOS, ALAT, triglycerides, total and HDL-cholesterol. Electrocardiogram (ECG) will also be measured based on medical history. Fasting plasma glucose value should be <7.0 mmol/l. If the 2-h glucose value > 11.0 mmol/l will be found in the OGTT in the beginning of the intervention, subject’s eligibility to continue in the study will be evaluated by a study physician.


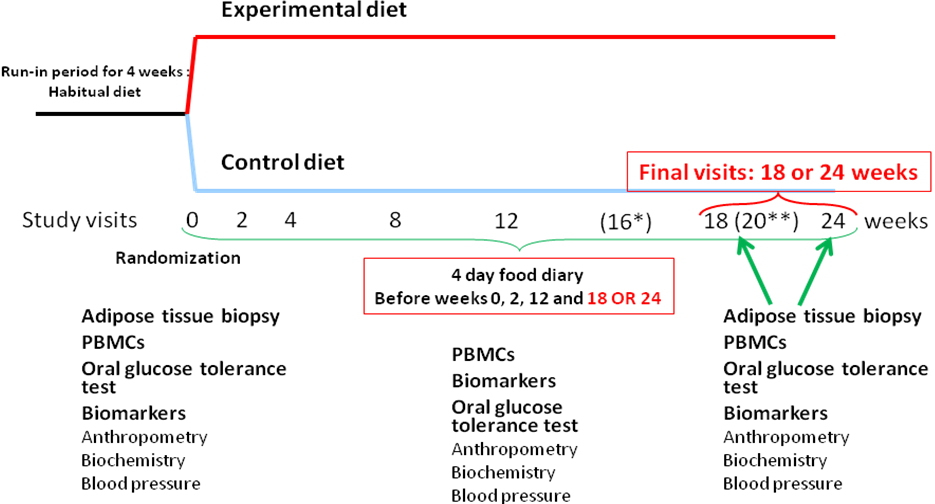


**Figure 2.** Study design in the diet intervention. PBMC= peripheral mononuclear cells.

* If the final visit will be done on the study week 18, the study visit on the week 16 is optional.

** Study visit at week 20 will be made if the intervention is planned to last 24 weeks, as originally decided.

**In two centres, Lund and Kuopio, the original study plan will be followed, whereas in all other centres the final study visit will happen at week 18. In statistical analyses regarding the major outcome measures this change is possible to take into account. It is expected that no major changes in variables of interest will any more happen between the weeks 18 and 24. The changes made in the study protocol should be informed appropriately. Of note, the trial has also been registered as a clinical trial (NCT00992641).**

- 1. **Study subjects**

Altogether **180 - 200 subjects** aged 30 to 65 years will be recruited from 6 centers (20-40 subjects/center) of the SYSDIET cohort. The inclusion criteria are listed in Table 2. The main inclusion criterion is BMI 27-38 kg/m^2^. The subjects should also have at least two other IDF criteria for metabolic syndrome listed in Table 1, except diagnosed type 2 diabetes. Persons will be screened mainly through advertising in newspapers, but also from previous clinical or epidemiological trials. The aim is to have male subjects at least 1/3 of the whole study group. Moreover, since the IDF criteria concern the Caucasian population, only Caucasians are included in the study. The use of anti-hypertensives and lipid lowering drugs should not be discontinued during the intervention. Based on the recent experiences from clinical trials and new power calculations 80-90 persons in each group will be expected to give appropriate power for the study (alfa level < 0.05, beta level 0.80).

**Table 2**. Inclusion criteria.

| Variable | Inclusion |
| --- | --- |
| Age | 30-65 years |
| BMI | 27-38 kg/m^2^ |
|  |  |
|  | + two other IDF criteria for metabolic syndrome (Table 1, except diagnosed type 2 diabetes) |
| Medication | Anti-hypertensives and lipid-lowering medication are allowed |

**3.3 Exclusion criteria**

The exclusion criteria are summarized in Table 3. The main exclusion criteria include any chronic disease and condition, which may hamper to follow dietary intervention protocol, poor compliance, chronic liver, thyroid and kidney diseases, alcohol abuse (> 40 g/d), known diabetes, fasting plasma glucose >7.0 mmol/l and BMI above 38 kg/m^2^.

Other exclusion criteria are a recent myocardial infarction (< 6 month), corticosteroid therapy, psychiatric disorders which need drug treatment, cancer under treatment, coeliac disease, allergies to cereals or fish and other serious and extensive food allergies. Exceptional diets (such as Atkin's or vegan diet) before the study are also an exclusion criterion as well as binge eating. Binge eating will be screened by using a validated questionnaire (Spitzer et al. 1993).

Inhaled corticosteroids are permitted. Fish and vegetable oil supplements should be discontinued at least 4 weeks before the beginning of the intervention (in the beginning of the run-in period). Moreover, the use of stanol or sterol esters should be discontinued in the beginning of the run-in period. Unwillingness to discontinue the use of these supplements and/or products is an additional exclusion criterion.

**Table 3**. Exclusion criteria

| Variable | Exclusion | Possibility for inclusion |
| --- | --- | --- |
| Fasting plasma glucose | > 7.0 mmol/l |  |
| Fasting plasma triglycerides | > 3.0 mmol/l |  |
| Fasting plasma total cholesterol | > 6.5 mmol/l |  |
| Blood pressure | > 160/100 mmHg | Should be stabilized with medication, after which the intervention can be started. |
| Preceding weight change | 5% or active weight loss during preceding 6 months |  |
| Chronic disease or condition | Any that may hamper the successful participation of the intervention (see background information form) |  |
|  | Liver disease |  |
|  | Kidney disease |  |
|  | Diabetes (both type 1 and type 2) |  |
| Thyroid disease | Newly found, unstabilized disease | Stabilized with medication |
| Myocardial infarction | Within previous 6 months |  |
|  |  |  |
|  |  |  |
|  |  |  |
| Alcohol abuse | > 40 g/day |  |
| Corticosteroid therapy | Oral medication | Inhaled, cream, lotion |
| Psychiatric disorders | Severe, with medication | ‘Mood’ medication |
| Cancer | Under treatment | Cured cancer allowed |
| Coeliac disease | Gluten free diet |  |
| Exceptional diets | Atkin’s, vegan, allergies to fish or cereals, other extensive allergies |  |
| Binge eating | Score of the BED in the QEWP questionniare |  |
| Fish and vegetable oil supplements | Unwillingness to discontinue the use of the supplements |  |
| Stanol and sterol esters | Unwillingness to discontinue the use of stanol/sterol containing products |  |

**3.3.1 Exclusion criteria during the intervention**

The participation of an uncompliant subject who repeatedly does not follow the instructions and does not consume the study products should be discontinued. The allowed weight change during the study is ± 2-3 %.

**3.4 Study diets**

The principles of the Exp-diet are summarized in Tables 4 and 5. Diet will be isocaloric based on the evaluation of the habitual diet made by a dietitian or a clinical nutritionist. The energy intake level of the diet will be planned based on the individual energy intake (calculated from food record) or estimated requirement. Mean total fat intake of 30-35 E% is divided into saturated fat intake ≤ 10 E% and mono- and polyunsaturated fat intake minimum 2/3 of total fat intake. Mean carbohydrate intake is 45-52 E% with the main emphasis to increase the use of whole grain cereal products of typical Nordic varieties such as rye, barley and oats. Intake of fruit, vegetables and berries should be ≥500 g. The aim for fiber intake is >35 g/d. Fish should be included in the diet >3 times (100-150 g /portion) per week of which 2 meals/week should be fatty fish. By choosing foods with low salt content the aim is to decrease salt intake to <7 g/d.

The food composition of the C-diet varies among Nordic countries, although the nutrient intake is close to each other in each country. The calculations of the dietary composition and mean nutrient intake in the C-diet are based on the mean intake figures in the five Nordic countries. Overall diet should be isocaloric and based on the evaluation of the habitual diet made by a dietitian or a clinical nutritionist. Differing from the Exp-diet, the mean intake of the total fat is about 35 E% divided into the intake of saturated fatty acids 15 E%, monounsaturated fatty acids 15 E% and polyunsaturated fatty acids 5 E%. Dietary fiber intake will be the typical Nordic intake of 15-20 g/d with habitual low consumption of fruits, vegetables and berries. The mean salt intake should be 10 g/day at its highest (Tables 4 and 5).

**Table 4**. Mean nutrient composition of the study diets*.

| **Nutrient** | **Experimental diet** | **Control diet** |
| --- | --- | --- |
| Carbohydrate (E%) | 45-52 | 45-47 |
| Sucrose (E%) | ≤ 10, max. 50 g/day | no restrictions |
| Fibre (g) | ≥ 35 g or 4g/MJ | 15-20 |
| Protein (E%) | 18-20 | 18-20 |
| Fat (E%) | 30-35 | 35 |
| Saturated (E%) | < 10 | 15 |
| Monounsaturated (E%) | minimum 2/3 of total fat intake | 15 |
| Polyunsaturated (E%) |  | 5 |
| Salt (g) | 6 g for women, 7g for men | ≤ 10 |

*Control diet is based on habitual diets in five Nordic countries with some modifications. Experimental diet is based on the Nordic recommendations.

**Table 5**. Typical food items in study diets.

| **Food group** | **Experimental diet** | **Control diet** |
| --- | --- | --- |
| Cereals | **≥ 25 % of total energy as whole grain:**  **of which ≥ 50 % as rye, barley and oat**  **Whole grain pasta and unpolished rice (≥ 6 g fiber/100 g) (≥ 2-3 meals/wk)**  **Low salt content (≤ 1.0 %) in breads is recommended**  **Cereals with no added sugar or honey are recommended**  **Bread (≥ 6 g fiber/100 g) ( ≥ 6 slices/day, 1 slice = 30-35 g)**   - e.g. whole grain rye, wheat, barley and oat bread, endosperm rye breads, ryebread (flat, boiled), sour dough bread, unsweetened bread with whole kernels, “pumpernickel”, crispbread (Knäckebröd) | **≥ 25 % of energy as refined:**  **of which ≥ 90 % as wheat**   - e.g. refined wheat bread, wheat oat bread (**≤** 5-6 g fiber/100g) - polished rice, refined wheat pasta - no whole kernels or rye sourdough |
| Vegetables etc. | **Fruits, vegetables and berries ≥ 500 g**   - **Berries** (**≥ 150-200 g/day)**   **variety of bilberries (e.g. whole berries, puree, berry powder), strawberries and one of the following, based on local preferences:** blueberries, black, red or white currants, raspberries, cherries, blackberries, gooseberries, elderberries, cloudberries, cowberries (wild)   - **Fruits** (**≥ 175 g/day)**   e.g. apples, pears, oranges, bananas, quince   - **Vegetables** (**≥ 175 g/day)**   e.g. tomato, cucumber, lettuce, carrot, leak, onion, rhubarb, sweet beet, turnip, parsnip, parsley, dill, radish, beetroot, chive, brassica [broccoli, Brussels sprout, cabbage (red and white), cale, cauliflower, swede], celery root  **Potatoes are not regarded as vegetable!** | **200 - 250 g**   - the same, but less   **(≤ 1 dl/50g /day)**  **Bilberries should**  **NOT be consumed**   - the same, but less - the same, but less |
| **Fats** | **Rapeseed oil**  **Rapeseed and/or sunflower oil and/or soyabean oil based margarines with no trans fatty acids and**  **≥ 2/3 of unsaturated fats**   - Nuts and seeds can be included in the diet, e.g. hazelnuts and unsalted and non-roasted sunflower seeds | **Butter or other milk fat based spread (≥ 50 % of total fat as saturated)** |
| **Milk products** | **Low fat liquid milk products including yoghurts, skyrs etc. (≤ 1 % of fat) Cheese (≤ 17 % of fat)**   - **≥ 2 portions/day  (1 portion= 2.5 dl of liquid dairy products or 2 slices of cheese)** - Sweetened yoghurts and other fruit milk products **should** be avoided | **No limitations** |
| **Fish** | **≥ 3 meals /week:**  **2 fatty fish (min. 4-5 % of fat) meals + 1 low fat fish meal**   - e.g. salmon, rainbow trout, Baltic herring, whitefish, vendace, pike, haddock, halibut, trout, herring, mackerel, cod, saithe, plaice, mackerel, sardines, brisling - Norway: herring and mackerel also available as spreads - Iceland: cod liver oil discontinued at least 4 weeks before the beginning of the intervention; replacement by pure D-vitamin supplements | **≤ 1 meal / week** |
| **Meat** | **Preferably white meat, poultry**  **Low fat choices**  **Game**   - e.g. poultry, lamb (lean products, **≤** 10 % of fat), game, pig (lean products, **≤** 10 %), beef (lean products, **≤** 10 %) | **No limitations**   - same, but less lean |
| **Soft drinks**  **Fruit and berry juices** | **Sugar-containing soft drinks should be avoided.**   - The use of artificially sweetened drinks should be limited to 1-2 glasses per day.   **≤ 1 glass (1.5 dl)/day**   - Finland: "National drink recommendation" allows 1-2 glasses of juices per day along with a meal and in the study diet sucrose limitation (max 50g/day) allows this, thus we see that 1 glass/day should be ok | **No limitations**  **No limitations** |

1. **Measurements**

The attached table (clinical, anthropometric and biochemical measurements) summarizes the main measurements that will be performed during the study. The methodology of analyses of the blood samples will be centralized concerning the non-rutine measurements and is based on the expertise of the partner center. These samples will, thus, be sent to these centers. Other measurements and analyses will be done locally in the performing centers according to the standard operational procedures to be accepted by the Management Group of the NCoE SYSDIET.

**Appendix 1 shows the participating centres and their responsibility regarding the intervention trial and analyses, which will be started in September 2010.**

**4.1 Biochemical and anthorometric measurements**

The separate SOPs are located in the intranet of the SYSDIET-project to specify the procedures on the biochemical and anthropometric measurements.

***Oral glucose tolerance test.*** In the beginning of the two-hour glucose tolerance test participants will drink 75 g of glucose/ 3 dl water. During the test blood samples will be taken at the time points 0, 30 and 120 minutes to measure the concentrations of glucose, insulin and triglycerides. PBMCs will be collected also at 0 and 120 min time points to follow an acute postprandial genetic response of these cells.

**4.2 Systems biology methodology**

It is now well known that dietary changes also induce changes in many biological phenomena, including gene expression, protein and lipid metabolism and different metabolic pathways. These phenomena will be studied applying modern high-throughput technologies of systems biology, such as transcriptomics, proteomics and metabolomics, especially lipidomics. Therefore, in addition to plasma samples we also collect adipose tissue and PBMCs samples as well as 24-h urine and faecal samples at the beginning and end of the study to search for early biomarkers (Figure 2) of disease pathogenesis and for the sensitivity to dietary modification.

**Figure 2.** Characterization of biomarkers in the different stages of the development of metabolic syndrome.

**Biomarker of an exposure**

**Biomarker of a target function**

**Biomarker of an endpoint**

**Improved**

**insulin sensitivity, blood pressure, lipid metabolism; adipokines, inflammation markers**

**Reduced risk of diabetes mellitus**

**and cardiovascular diseases**

**Dietary modification**

**Tissue gene expression, protein activation, function and tissue metabolism**

**Insulin resistance**

**Metabolic**

**syndrome**

**Abdominal obesity**

**4.3 Genetic samples**

Adipose tissue samples will be taken from the subcutaneous adipose tissue before and after the intervention. Samples will be taken as a needle biopsy under local anaesthesia (Lidocain ® without adrenalin) from the midpoint of suprailiaca and umbilicus. Right after the biopsy, samples will be washed twice to remove blood contamination and immerse to liquid nitrogen. After this, samples will be stored at -80ºC until analysed.

The blood samples will be taken to separate PBMCs for studying mRNA expression before and after the intervention. PBMCs will be separated with special tubes developed for the purpose (BD vacutainer CPT, Cell Preparation Tubes, Beckton Dickinson). Separated PBMCs will be suspended to lysing buffer and stored -80ºC.

Total RNA will be isolated using commercial kits. After the RNA isolation complementary DNA (cDNA) synthesis will be performed using commercial kits. cDNA will be labelled, fragmented and hybridised at the microarray chip (15 subjects/study group). qPCR-analysis will be done for all the study subjects. Genes that have been previously associated with glucose-, lipid-, energy- and adipose tissue metabolism will be investigated using qPCR method. Additional analyses will be done for some potentially interesting genes from our previuos transcriptomic analysis.

***Genetics.*** Based on results from transcriptomics, presviously known and unknown genes will be found to respond to dietary modification. In this study, the emphasis of the genetic analysis will be on studying their sequence variations, mutations and functional studies. In addition, variation of genes previously associated with energy- glucose- and adipose tissue metabolism and with inflammation will be studied. Specifically the interest will be on the metabolic pathways that have been associated to the development and/or risk of features of metabolic syndrome, T2DM and CVD. Genes linked to new pathways based on our previous transcriptomic analysis that are important for the development of the conditions mentioned above will be studied, too. The results of these analysis will be explored in the light of the results from clinical and biochemical measurements.

Blood sample for the isolation of DNA will be taken at the beginning of the intervention. DNA will be isolated from white blood cells. The blood samples will be stored at –80ºC until isolated and analysed. DNA-samples will be stored at +4ºC. The sequence variations of the gens will be analysed using TaqMan chemistry (Applied Biosystems). The sequences and known variations will be search from the internet databases (HAPMAP-database, NCBI, UCSC, ENSEMBLE). If variations are not known for particular gene, it will be sequenced using DNA-samples from about 40 subjects. The found variations will then be analysed from the whole study population.

**5. Ethical aspects**

**5.1 The information to be given to subjects**

The study aims, design and progression and the content of study visits will be discussed with study subjects in detail. They will also receive written information these aspects. Subjects have to sign the written consent separately for the different parts of study (dietary intervention, genetic analysis (DNA and gene expression separated), donation of faecal and urine samples). Subjects have every right to discontinue the study without telling the possible reason for it. If the disease needing treatment will be diagnosed, particular subject will be referred to approapriate medical treatment.

Study subjects will receive all the information from the clinical and biochemical measurements done during the intervention. The genetic information will not be told to study subjects. The reason for this is that one particular gene or its manner of expression does not cause the development of any feature of metabolic syndrome or chronic disease of interets (T2DM, CVD). The genes of interest are only predisposing factors for the disease. In this kind of study, the clinical meaning of the variation of single gene is impossible to evaluate. DNA- and RNA- samples are not used for diagnostic tests or tools. Moreover, the interpretation of the results at the individual level is hard if not impossible. Thus, genetic information will be studies and analysed only at the group level. If somebody of the subjects insists information of his/her own genetic factors that has been analysed in this study, the researcher will discuss with him/her about these results and their possible clinical meaning in the light of the current literature and knowledge.

**5.2 Costs to be covered for the participants**

Study subjects will receive information of the costs covered by the project. The main principle is that only the travel costs are covered by the level of the public transportation. Some costs for buying the foods to carry out the intervention are covered: for example the costs of consuming fish will be covered by the receipts. Other key food items will be delivered from the University facilities, and do not cause any extra costs.

#### 5.3 Risks

There are no significant risks to participate in the present study. Diet will be composed of the typical Nordic food items. Clinical nutritionist will give the individual advice on how to follow the study diet. The well-being of the subjects will be followed during the study visits. They also have a possibility to contact personel outside the office hours if needed. The blood and tissue samples are taken during the intervention. The blood samples will be taken by the experienced laboratory nurse. Medical doctor will take the adipose tissue sample. If some complications appear, the costs will be covered by the research project.

**5.4 Anonymity of the samples**

The samples will be coded so that only study nurse or responsible researcher /centre are able to combine the results to the individual subject. The samples are coded with study ID, usually number that is used in all samples and measurements of the particular subject. The results are confidential and they are not given to outsiders.

**7. Publications**

The main results will be published in top level scientific journals. In addition, this study will produce large amount of new data on the field of nutrigenetics, nutrigenomics, metabolomics and systems biology to be published according to the agreements among PIs of the consortium.

**8. Discussion**

One of the biggest challenges of SYSDIET is to build up a Nordic platform for controlled dietary interventions. This study protocol and the main objectives form the first step in this regard. Based on the former experiences we believe that this study protocol is realistic and it will give new results as for what is the role of qualitative changes in Nordic diet in the development of and consequently prevention and treatment of metabolic syndrome.

The changes in the study protocol or the shorter period of the intervention do not violate the main purpose of the study or its main objectives. Based on new power calculations and recent experiences from other dietary intervention trials show that 80 to 90 persons per intervention group is enough to show significant differences in major outcome measures.

**9. Nordic Centre of Exellence SYSDIET partners participating in the carrying out the intervention:**

***DENMARK:***

Aarhus University Hospital, Department of Endocrinology and Metabolism:

Prof Kjeld Hermansen ([kjeld.hermansen@aarhus.rm.dk](mailto:kjeld.hermansen@aarhus.rm.dk) or [kjeld.hermansen@mail.dk](mailto:kjeld.hermansen@mail.dk))

***FINLAND:***

University of Kuopio (COORDINATION), Departments of Clinical Nutrition and Biochemistry:

Prof. Matti Uusitupa (matti.uusitupa@uef.fi), PhD, Doc. Marjukka Kolehmainen ([marjukka.kolehmainen@uef.fi](mailto:marjukka.kolehmainen@uef.fi)), Prof Kaisa Poutanen(kaisa.poutanen@vtt.fi), PhD, Doc. Ursula Schwab ([ursula.schwab@uef.fi](mailto:ursula.schwab@uef.fi)),

University of Oulu, Department of Medicine: Prof Markku Savolainen, Prof Karl-Heinz Herzig

***ICELAND:***

University of Iceland, Unit for Nutrition Research:

Prof Inga Thorsdottir ([ingathor@landspitali.is](mailto:ingathor@landspitali.is))

***SWEDEN:***

Uppsala University/ Clinical Nutrition and Metabolism:

Prof Ulf Risérus (ulf.riserus@pubcare.uu.se)

Lund University, Biomedical nutrition, Center for Chemistry and Chemical Engineering, Lund Institute of Technology:

Prof Björn Åkesson (Bjorn.Akesson@kc.lu.se), Prof Gunilla Önning (Gunilla.Onning@kc.lu.se)

**Literature**

Bergman RN. Toward physiological understanding of glucose tolerance. Minimal-Model approach. Diabetes 1989;38:250-256.

Curtis K, Orešič M, Vidal-Puig A. Pathways to analysis of microarray data. Trends Biotechnol 2005;8: 429-435.

Finnegan YE, Minihane AM, Leigh-Firbank EC, Kew S, Meijer GW, Muggli R, Calder PC, Williams CM. Plant- and marine-derived n-3 polyunsaturated fatty acids have differential effects on fasting and postprandial blood lipid concentrations and on the susceptibility of LDL to oxidative modification in moderately hyperlipidemic subjects. Am J Clin Nutr 2003;77:783-795.

Gopalacharyulu PV, Lindfors E, Bounsaythip C, Kivioja T, Yetukuri L, Hollmén J, Orešič M. Data integration and visualization system for enabling conceptual biology. Bioinformatics 2005;21:i177-i185.

Grundy SM. Metabolic syndrome: connecting and reconciling cardiovascular and diabetes worlds.
J Am Coll Cardiol 2006;47:1093-1100.

Hu FB, Willett WC Optimal diets for prevention of coronary heart disease. JAMA 2002;288:2569-2578.

Jayaprakasam B, Vareed SK, Olson LK, Nair MG. Insulin secretion by bioactive anthocyanins and anthocyanidins present in fruits. J Agric Food Chem 2005;53:28-31.

Juntunen KS, Laaksonen DE, Autio K, Niskanen LK, Holst JJ, Savolainen KE, Liukkonen KH, Poutanen KS, Mykkänen HM. Structural differences between rye and wheat breads but not total fiber content may explain the lower postprandial insulin response to rye bread. Am J Clin Nutr 2003;78:957-964.

Katajamaa M, Orešič M. Processing methods for differential analysis of LC/MS profile data. BMC Bioinformatics 2005;6:179(1-12).

Knowler WC, Barrett-Connor E, Fowler SE, Hamman RF, Lachin JM, Walker EA, Nathan DM. Diabetes Prevention Program Research Group. Reduction in the incidence of type 2 diabetes with lifestyle intervention or metformin. N Engl J Med 2002;346:393-403.

Kolehmainen M, Uusitupa MIJ, Alhava E, Laakso M, Vidal H. Effect of the Pro12Ala polymorphism in the peroxisome proliferator-activated receptor γ2 gene in the regulation of PPARγ target genes expression in adipose tissues of massively obese subjects. J Clin Endocrinol Metab 2003;88:1717-1722.

Kuusisto J, Mykkänen L, Pyörälä K, Laakso M. NIDDM and its metabolic control predict coronary heart disease in elderly subjects. Diabetes 1994;43:960-967.

[Laaksonen DE, Lakka HM, Niskanen LK, Kaplan GA, Salonen JT, Lakka TA.](http://www.ncbi.nlm.nih.gov/entrez/query.fcgi?cmd=Retrieve&db=pubmed&dopt=Abstract&list_uids=12446265&query_hl=11&itool=pubmed_docsum) Metabolic syndrome and development of diabetes mellitus: application and validation of recently suggested definitions of the metabolic syndrome in a prospective cohort study. Am J Epidemiol 2002;156:1070-1077.

Laaksonen DE, Niskanen L, Lakka HM, Lakka TA, Uusitupa M. Epidemiology and treatment of the metabolic syndrome. Ann Med 2004;36:332-346.

Laaksonen DE, Toppinen LK, Juntunen KS, Autio K, Liukkonen KH, Poutanen KS, Niskanen L, Mykkänen HM. Dietary carbohydrate modification enhances insulin secretion in persons with the metabolic syndrome. Am J Clin Nutr 2005;82:1218-1227.

Lafontan M, Arner P. Application of in situ microdialysis to measure metabolic and vascular responses in adpose tissue. Trends Pharmac Sci 1996;17:309-313.

Lakka HM, Laaksonen DE, Lakka TA, Niskanen LK, Kumpusalo E, Tuomilehto J, Salonen JT. The metabolic syndrome and total and cardiovascular disease mortality in middle-aged men. JAMA 2002;288:2709-2716.

Leigh-Firbank EC, Minihane AM, Leake DS, Wright JW, Murphy MC, Griffin BA, Williams CM. Eicosapentaenoic acid and docosahexaenoic acid from fish oils: differential associations with lipid responses. Br. J. Nutr. 2002;87:435-45.

Lichtenstein AH, Schwab US. Relationship of dietary fat to glucose metabolism. Atherosclerosis 2000;150:227-243.

Lindström J, Peltonen M, Eriksson JG, Louheranta A, Fogelholm M, Uusitupa M, Tuomilehto J. High-fibre, low-fat diet predicts long-term weight loss decreased type 2 diabetes risk: the Finnish Diabetes Prevention Study. Diabetologia 2006;49:912-920.

McCarthy MI. Progress in defining the molecular basis of type 2 diabetes mellitus through susceptibility-gene identification. Hum Mol Genet 2004;13, Review issue 1:R33-R41.

McDougall GJ, Shpiro F, Dobson P, Smith P, Blake A, Stewart D. Different polyphenolic components of soft fruits inhibit α-amylase and α-glucosidase. J Agric Food Chem 2005;53:2760-2766.

Medina-Gomez G, Virtue S, Lelliott C, Boiani R, Campbell M, Christodoulides C, Perrin C, Jimenez-Linan M, Blount M, Dixon J, Zhan D, Thresher RR, Aparicio S, Carlton M, Colledge WH, Kettunen MI, Seppänen-Laakso T, Sethi JK, O’ Rahilly S, Brindle K, Cinti S, Oresic M, Burcelin R, Vidal-Puig A. The link between nutritional status and insulin sensitivity is dependent on the adipocyte-specific PPAR gamma 2 isoform. Diabetes 2005;54:1706-1716.

Mori TA, Bao DQ, Burke V, Puddey IB, Watts GF, Beilin LJ. Dietary fish as a major component of a weight-loss diet: effect on serum lipids, glucose, and insulin metabolism in overweight hypertensive subjects. Am J Clin Nutr 1999;70:817-825.

Montonen J, Knekt P, Järvinen R, Aromaa A, Reunanen A. Whole-grain and fiber intake and the incidence of type 2 diabetes. Am J Clin Nutr 2003;77:622-629.

Murtaugh MA, Jacobs DR Jr, Jacob B, Steffen LM, Marquart L. Epidemiological support for the protection of whole grains against diabetes. Proc Nutr Soc 2003;62:143-149.

Pan XR, Li GW, Hu YH, Wang JX, Yang WY, An ZX, Hu ZX, Lin J, Xiao JZ, Cao HB, Liu PA, Jiang XG, Jiang YY, Wang JP, Zheng H, Zhang H, Bennett PH, Howard BV. Effects of diet and exercise in preventing NIDDM in people with impaired glucose tolerance. The Da Qing IGT and Diabetes Study. Diabetes Care 1997;20:537-544.

Peltonen M, Korpi-Hyövälti E, Oksa H, Puolijoki H, Saltevo J, Vanhala M, Saaristo T, Saarikoski L, Sundvall J, Tuomilehto J. Lihavuuden, diabeteksen ja muiden glukoosiaineenvaihdunnan häiriöiden esiintyvyys suomalaisessa aikuisväestössä Dehkon 2D-hanke (D2D). SLL 2006;3:163–170.

Ramachandran A, Snehalatha C, Mary S, Mukesh B, Bhaskar AD, Vijay V. Indian Diabetes Prevention Programme (IDPP). The Indian Diabetes Prevention Programme shows that lifestyle modification and metformin prevent type 2 diabetes in Asian Indian subjects with impaired glucose tolerance (IDPP-1). Diabetologia 2006;49:289-297.

Reaven GM. Banting lecture 1988. Role of insulin resistance in human disease. Diabetes 1988;37:1595-1607.

Sarkkinen E, Korhonen M, Erkkilä A, Ebeling T, Uusitupa M. Effect of apolipoprotein E polymorphism on serum lipid response to the separate modification of dietary fat and dietary cholesterol. Am J Clin Nutr 1998;68:1151-1152.

Schwab US, Ågren JJ, Valve R, Hallikainen MA, Sarkkinen ES, Jauhiainen M, Karvonen MK, Pesonen U, Koulu M, Uusitupa M, Savolainen MJ. The impact of the leucine 7 to proline 7 polymorphism of the neuropeptide Y gene on postprandial lipemia and on the response of serum total and lipoprotein lipids to a reduced fat diet. Eur J Clin Nutr 2002;56:149-156.

Sirtori CR, Crepaldi G, Manzato E, Mancini M, Rivellese A, Paoletti R, Pazzucconi F, Pamparana F, Stragliotto E. One-year treatment with ethyl esters of n-3 fatty acids in patients with hypertriglyceridemia and glucose intolerance. Reduced triglyceridemia, total cholesterol and increased HDL-C without glycemic alterations. Atherosclerosis 1998;137:419-427.

Spitzer RL, Yanovski S, Wadden T, Wing R, Marcus MD, Stunkard A, Devlin M, Mitchell J, Hasin D, Horne RL. Binge eating disorder: Its further validation in a multisite study. Int J Eat Disord 1993;13:137-153.

Tsuda T, Ueno Y, Aoki H, Koda T, Horio F, Takahashi N, Kawada T, Osawa T. Anthocyanin enhances adipocytokine secretion and adipocyte-specific gene expression in isolated rat adipocytes. Biochem Biophys Res Commun 2004;316:149-157.

Tsuda T, Ueno Y, Kojo H, Yoshikawa T, Osawa T. Gene expression profile of isolated rat adipocytes treated with anthocyanins. Biochim Biophys Acta 2005;1733:137-147.

Tsuda T, Ueno Y, Yoshikawa T, Kojo H, Osawa T. Microarray profiling of gene expression in human adipocytes in response to anthocyanins. Biochem Pharmacol 2006;71:1184-1197.

Tuomilehto J, Lindström J, Eriksson JG, Valle TT, Hämäläinen H, Ilanne-Parikka P, Keinänen-Kiukaanniemi S, Laakso M, Louheranta A, Rastas M, Salminen V, Uusitupa M. Finnish Diabetes Prevention Study Group. Prevention of type 2 diabetes mellitus by changes in lifestyle among subjects with impaired glucose tolerance. N Engl J Med 2001;344:1343-1350.

Uusitupa M. Gene-diet interaction in relation to the prevention of obesity and type 2 diabetes: evidence from the Finnish Diabetes Prevention Study. Nutr Metab Cardiovasc Dis 2005;15:225-233.

Uusitupa M, Lindi V, Louheranta A, Salopuro T, Lindström J, Tuomilehto J; Finnish Diabetes Prevention Study Group. Long-term improvement in insulin sensitivity by changing lifestyles of people with impaired glucose tolerance: 4-year results from the Finnish Diabetes Prevention Study. Diabetes 2003;52:2532-2538.

Uusitupa MI, Niskanen LK, Siitonen O, Voutilainen E, Pyörälä K. Ten-year cardiovascular mortality in relation to risk factors and abnormalities in lipoprotein composition in type 2 (non-insulin-dependent) diabetic and non-diabetic subjects. Diabetologia. 1993;36:1175-1184.

Vessby B, Uusitupa M, Hermansen K, Riccardi G, Rivellese AA, Tapsell LC, Nalsen C, Berglund L, Louheranta A, Rasmussen BM, Calvert GD, Maffetone A, Pedersen E, Gustafsson IB, Storlien LH; KANWU Study. Substituting dietary saturated for monounsaturated fat impairs insulin sensitivity in healthy men and women: The KANWU Study. Diabetologia. 2001;44:312-319.

Woodman RJ, Mori TA, Burke V, Puddey IB, Watts GF, Beilin LJ. Effects of purified eicosapentaenoic and docosahexaenoic acids on glycemic control, blood pressure, and serum lipids in type 2 diabetic patients with treated hypertension. Am. J. Clin. Nutr. 2002;76:1007-1015.

Yudkin JS. Adipose tissue, insulin action and vascular disease: inflammatory signals. Int J Obes Relat Metab Disord 2003;27 (Suppl 3):S25-S28.

**Appendix 1. The centres participating in carrying out the SYSDIET intervention or are responsible for certain centralised analyses. Centralised analyses will be started in September 2010 according to the revised protocol.**

| Centre | Sysdiet intervention | Local analyses | Centralised analyses | Comments |
| --- | --- | --- | --- | --- |
| Lund University | 40 | Yes | beta- and alfa-carotenes |  |
| University of Eastern Finland | 35 | Yes | cytokines and adipokines, DNA-analyses: genotyping |  |
| University of Oulu | 40 | Yes | NMR-lipidomics |  |
| Aarhus University Hospital | 40 | Yes | serum 25-OH vitamin D, parathyroid hormone |  |
| University of Iceland | 20 | Yes | - |  |
| Uppsala University | 10-20 | Yes | serum fatty acid composition, alkylresorcinols |  |
| Karolinska Institute | - | - | adipose tissue analyses including transcriptomics, epigenetics |  |
| University of Copenhagen | - | - | plasma isoprostanes, metabolomics: plasma phytochemicals, urine metabolomics, faecal water soluble metabolites |  |
| VTT, Espoo | - | - | centralised data management and analyses, plasma lipidomics, global metabolomics |  |
| Akershus University Collage | - | - | PBMC analyses including transcriptomics |  |
| Nofima Food | - | - | Microbiota analyses from faeces |  |
| **IN TOTAL** | **185-195** |  |  |  |

Prof Kim Overvad and Prof Kund Erik Bach Knudsen from Aarhus University are also partners of SYSDIET.
